# Emperor Penguins Breeding on Iceshelves

**DOI:** 10.1371/journal.pone.0085285

**Published:** 2014-01-08

**Authors:** Peter T. Fretwell, Phil N. Trathan, Barbara Wienecke, Gerald L. Kooyman

**Affiliations:** 1 British Antarctic Survey, Cambridge, United Kingdom; 2 Australian Antarctic Division, Hobart, Tasmania, Australia; 3 Scripps Institution of Oceanography, University of California San Diego, La Jolla, California, United States of America; University of Lleida, Spain

## Abstract

We describe a new breeding behaviour discovered in emperor penguins; utilizing satellite and aerial-survey observations four emperor penguin breeding colonies have been recorded as existing on ice-shelves. Emperors have previously been considered as a sea-ice obligate species, with 44 of the 46 colonies located on sea-ice (the other two small colonies are on land). Of the colonies found on ice-shelves, two are newly discovered, and these have been recorded on shelves every season that they have been observed, the other two have been recorded both on ice-shelves and sea-ice in different breeding seasons. We conduct two analyses; the first using synthetic aperture radar data to assess why the largest of the four colonies, for which we have most data, locates sometimes on the shelf and sometimes on the sea-ice, and find that in years where the sea-ice forms late, the colony relocates onto the ice-shelf. The second analysis uses a number of environmental variables to test the habitat marginality of all emperor penguin breeding sites. We find that three of the four colonies reported in this study are in the most northerly, warmest conditions where sea-ice is often sub-optimal. The emperor penguin’s reliance on sea-ice as a breeding platform coupled with recent concerns over changed sea-ice patterns consequent on regional warming, has led to their designation as “near threatened” in the IUCN red list. Current climate models predict that future loss of sea-ice around the Antarctic coastline will negatively impact emperor numbers; recent estimates suggest a halving of the population by 2052. The discovery of this new breeding behaviour at marginal sites could mitigate some of the consequences of sea-ice loss; potential benefits and whether these are permanent or temporary need to be considered and understood before further attempts are made to predict the population trajectory of this iconic species.

## Introduction

Recent studies suggest that emperor penguin populations will decline in future decades due to climate change [1]–[6]. Current projections suggest that the world population will halve before 2052[3] with more northerly colonies, above 70°S being lost entirely [6]. This has led the IUCN to re-list the species from “Least Threatened” to “Near Concern” [7]. The primary reason cited for this predicted decline is the species’ reliance on sea-ice, a habitat that is expected to decrease in future years [8], [9]. Sea ice is important to the species in two ways; firstly as a breeding platform and secondly as a foraging environment. A decrease in sea-ice distribution will negatively impact food webs [10], reducing numbers of Krill (*Euphausia superb), and the higher trophic levels which feed on Krill such as glacial squid* (*Pleuragramma antarcticum*); two species which compose the majority of the emperors diet [11]. A decrease in food availability may negatively affect survival, breeding success, recruitment and therefore population size.

As a breeding platform, stable or “fast” sea-ice is required which forms when the emperors arrive at their breeding locations (usually in April) and remains unbroken until the chick fledge (usually in December). If the sea-ice breaks up too early in the season it will result in high chick mortality [1]–[6], multiple years of poor sea-ice will lead to poor breeding success, population decline and eventual extinction of a colony [12]. The emperor penguin is a sea-ice obligate, the species is too clumsy to climb onto ice shelves and needs ice of a low freeboard to exit the ocean [2]. Of the 46 colonies presently known 44 breed on fast-sea- ice [13] (stable sea-ice attached to the coast). Of the two remaining colonies, one is recorded as breeding on rock and one on a frozen lake, both of these colonies are small, one having a recorded population of 2900 pairs and the other 250 pairs [13] (the mean colony size is approximately 5500 pairs).

In recent years satellite observations have improved our knowledge of the emperor penguins breeding distribution [14] and population [13]. Here we report on newly discovered breeding behaviour in emperor penguins seen from satellite and aerial surveys. Four emperor colonies have been observed breeding on ice-shelves not sea-ice The first, discovered in 2009[15]on the West Ice Shelf at the edge of Barrier Bay was a small colony of that could have been judged an anomaly or a break off group from the larger West Ice shelf colony located ∼110 km to the north. However, since the discovery of the colonies on the West Ice Shelf, three other, large colonies, have been found that are either permanently, or annually located on ice shelves rather than on sea-ice.

Whereas sea-ice is frozen sea-water, ice-shelves are floating glacial ice that has flowed from the land into the sea; where the base of such glaciers breaks hydrostatic equilibrium, the ice-foot detaches from the ground bed and the glacial ice floats. When a single glacier feeds into the sea a glacier tongue is formed, but around the Antarctic coastline it is more common for ice from several glaciers or ice-streams to merge to form an ice-shelf. At their terminus ice-shelves can form ice cliffs, in some place over 60 metres high, although a few tens of metres is more common. Ice creeks often indent the cliff face giving a potential route up onto the ice-shelf itself, or where ice shelves are ablating the ice cliff may be less steep. As sea-ice forms, local weather conditions mean it can be highly variable in extent and duration, and therefore highly susceptible to regional climate change [8]. Ice-shelves are less dynamic, and are less susceptible to weather patterns and storm events, although cyclical calving events could pose a threat to organisms located near the ice-cliff edge and over longer time periods ice-shelves can collapse catastrophically such as the well documented break-up of the Larsen B Ice Shelf in 2002[16].

It is at present unclear whether this behaviour of breeding on ice shelves is a new phenomenon associated with recent climate change, or one that has always existed but has not yet been documented. Models of how animals adapt to climatic change exist [17] and we examine how this phenotype plasticity fits into those theories (see discussion).

That emperor penguins can move their breeding site depending upon ice conditions to a more stable location, including onto the top of the ice-shelf itself, means new factors should be incorporated into modelled population trajectories for this species. Whether such factors will provide temporary or permanent relief from the impacts of climate change remains uncertain.

The fact that emperors exhibit a previously unknown breeding behaviour, intimates that other less-well known species may also have similar unknown adaptive behaviours that may also offer temporary or permanent relief to the challenges of climate change.

## Materials and Methods

### Observations

**Figure 1:**
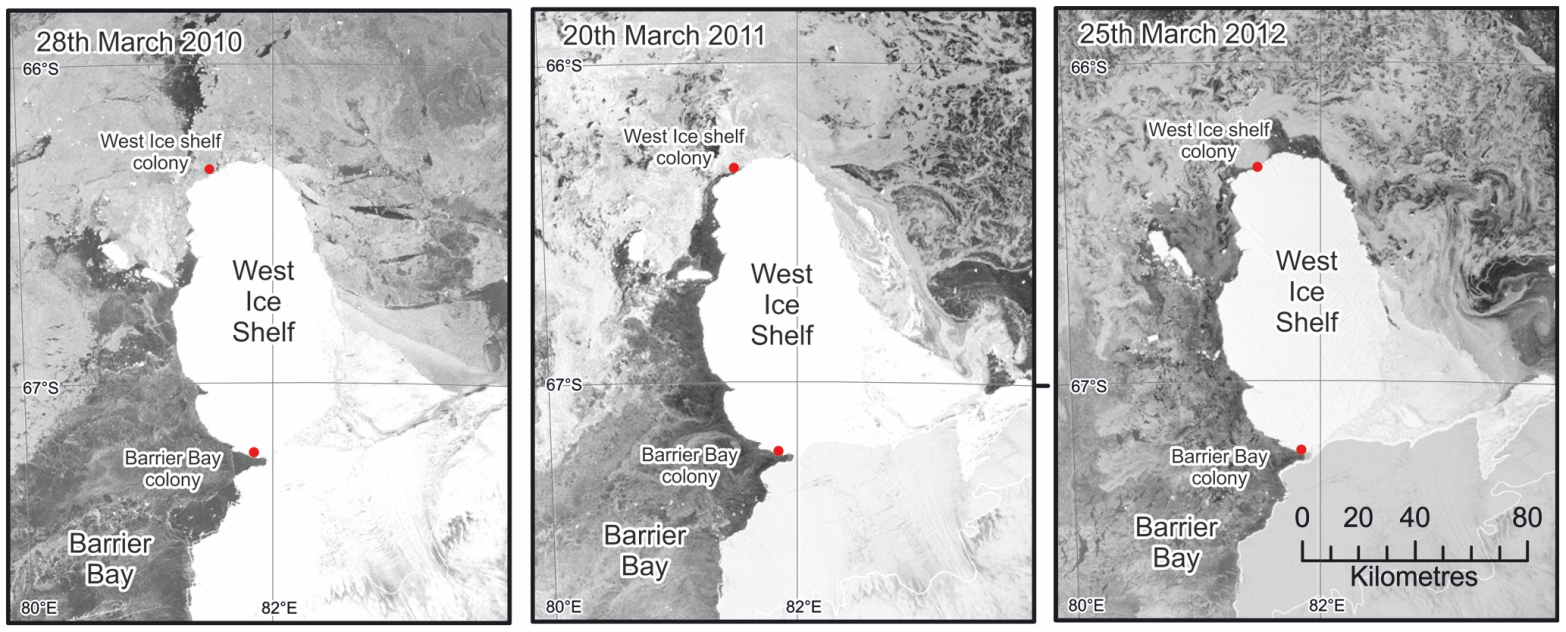
Envisat images showing the sea-ice conditions in late March around the West Ice shelf where two emperor colonies are located (equivalent imagery for 2009 is not available). Darker areas denote poor sea-ice, grey shows thicker sea-ice and white indicates ice shelf. Note that the Barrier Bay colony has a permanent polynya while the West Ice shelf colony located on the sea-ice has thicker sea-ice at this time of year when the birds would be arriving in the area to breed (images courtesy of Polarview – www.polarview.aq).

The first emperor colony found on an ice shelf was the Barrier Bay colony, 67.22°S, 81.93°E discovered in December 2009 [14]. Some 295 chicks and a small number of adults were seen. The group was located near the edge of the ice cliff of the West Ice Shelf. The birds had accessed the ice shelf via an ice gully approximately 5km to the southeast. The colony has been observed in three subsequent years in the same position. The presence of chicks at Barrier Bay confirms that this is a breeding location rather than a temporary site (Figure 1).

**Figure 2:**
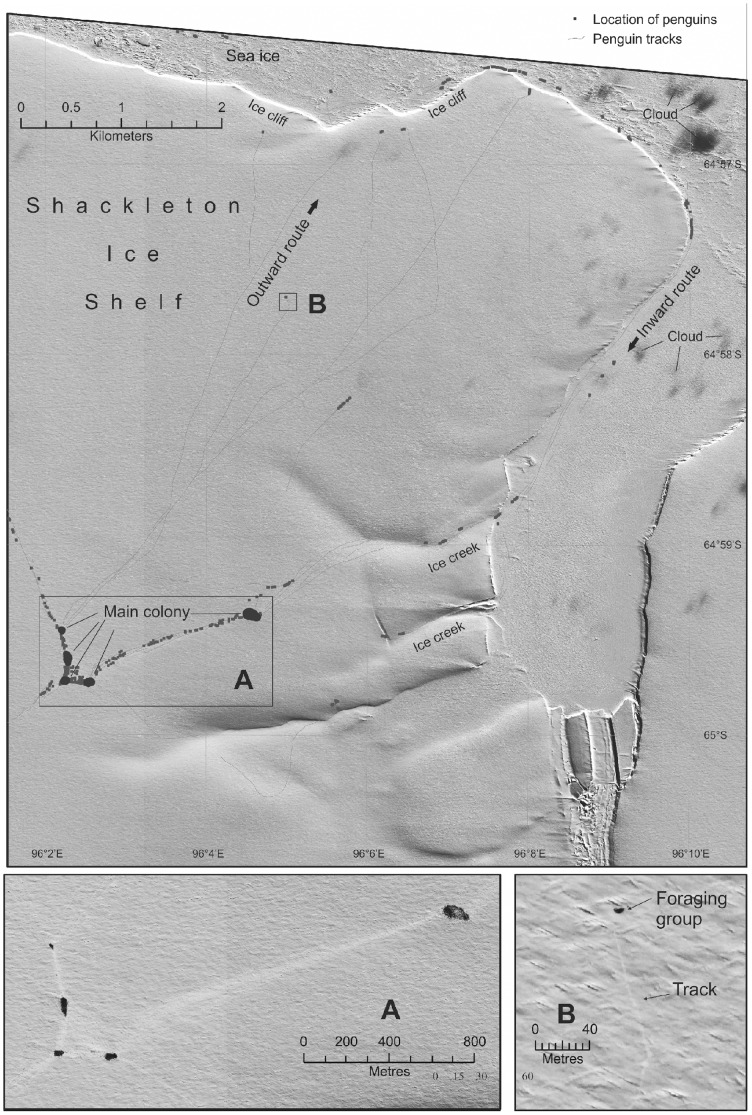
Shackleton Ice Shelf very high resolution satellite image. WorldView2 image (15 September 2012) showing the location of the Shackleton Ice shelf colony in 2012 in context with the ice edge. On this image the four main sub-colonies are clearly visible on top of the Shackleton Ice Shelf around 5 km from the ice cliffs that form the edge of the ice shelf. The image data also clearly shows groups of penguins on their way to and from the ice edge, and the tracks they leave behind them. By marking these trails it is possible to assess where each group has come from and which direction it is heading. Interestingly at this site, the outgoing forages use a different route to the incoming penguins; on the way out they negotiate a large ice cliff. ICESAT data from the area suggest that the top of the ice sheet is 32–34 m high, the image shows no slope down to the cliff so the drop may be considerable (although there is evidence of large snowdrifts abutting the cliff). The incoming parties cannot negotiate the cliff and so take a 5 km longer route around the edge of the ice shelf until they can access the gentle slopes afforded by a number of ice creeks to the east of the colony. How the emperor penguins get down the ice cliff is, at present, unclear.

The second colony identified on the top of an ice shelf is the Shackleton Ice Shelf colony. This colony was first located in 2008 [14] at 64.86°S, 96.02°E. The December 2008 position, found by Landsat imagery and later confirmed by Very High Resolution (VHR) satellite imagery (November 2009), was on sea-ice. The colony comprised approximately 6,470 pairs [13]; satellite observations confirmed that the breeding location remained constant in 2008, 2009 and 2010. However, in 2011 it appeared 15km to the south (64.98°S, 96.06°E) of its original location and on top of the ice shelf. The access route to the top of the shelf was a gulley 3.4 km to the east. In 2012, the breeding location was the same as in 2011 (Figure 2).

The third breeding location on top of an ice shelf is near the Jason Peninsula, at the northern limit of the Larsen C Ice Shelf. In 1893, the explorer and sealer Carl Anton Larsen was the first to visit this area [18], [19]. He reported on 4 December 1893 that “The kongepenguinerne (king penguin) are very numerous in those (ice) fjords” (ice creeks are a favoured breeding location of emperor penguin colonies). When recording this, his ship was located on the northern side of what became known as the Larsen C Ice Shelf (noon position of 67.00°S, 60°.00W). At the time of this discovery little was known about emperor penguins and they were often confused with the similar, but smaller king penguin (*A. patagonicus*), a species which Larsen would have been familiar with from his sealing trips to South Georgia. It is likely that Larsen’s sighting late in the breeding season indicated a colony in the vicinity. Although exhaustive satellite searches of the sea-ice in the area during previous studies [14], [15] were conducted, no colony was found.

**Figure 3:**
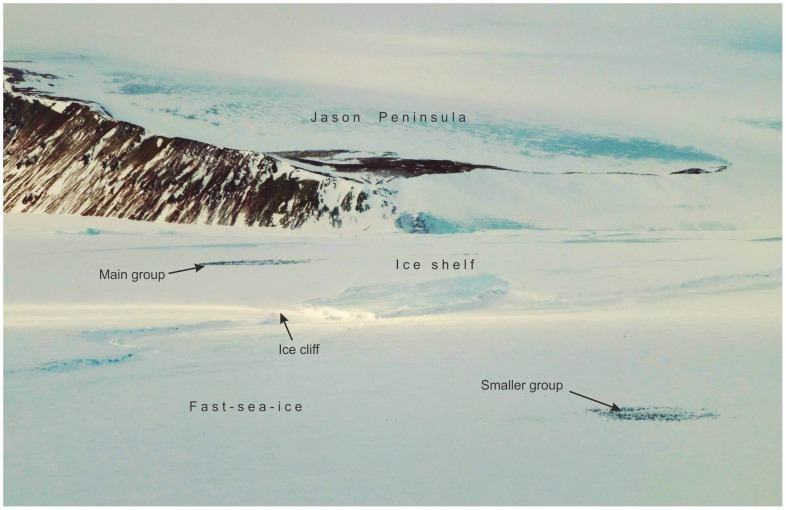
Emperor penguins on the edge of the Larsen Ice Shelf near the Jason Peninsula late in the breeding season. The larger group is on the ice-shelf, the smaller group has moved onto the fast-ice; earlier in the season data from QuickBird1 satellite imagery shows that the whole colony was located on the ice-shelf. Note the ice cliff which is probably an insurmountable barrier to the adult emperor penguins. No evident route to the colony was determined from the images. (Photo Ian Potten).

**Figure 4:**
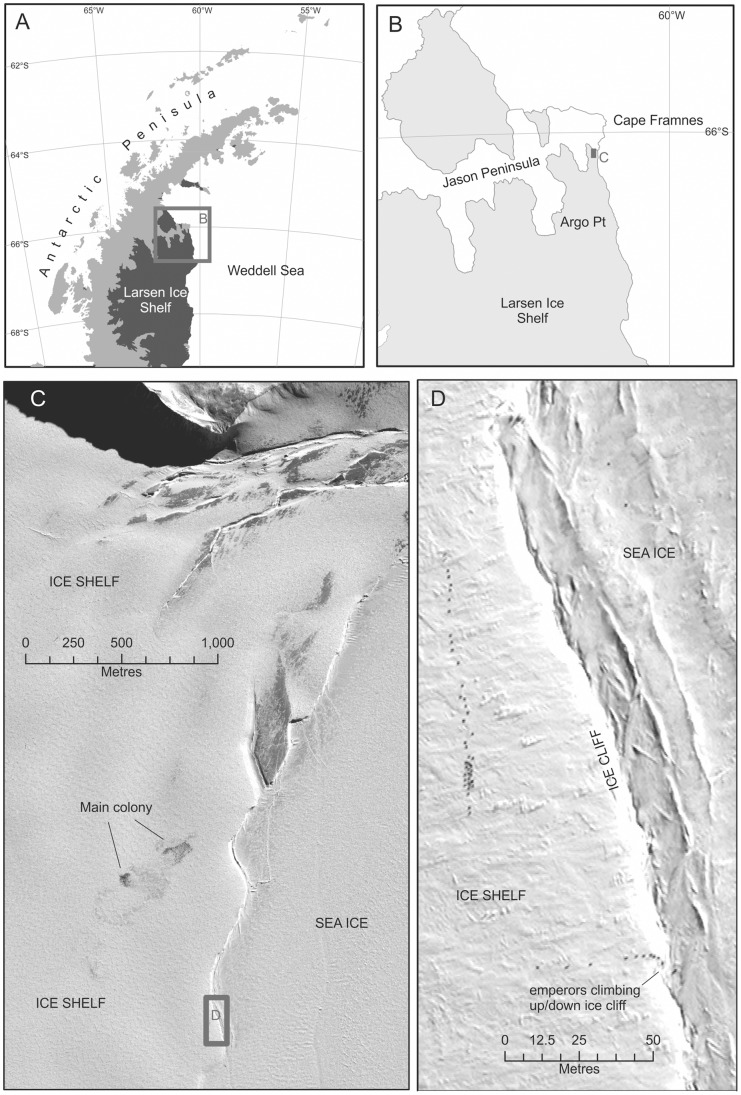
A and B; maps showing location of the Jason Peninsula colony. C: Quickbird1 image (12 Sept 2012) showing the main Jason Peninsula emperor penguin colony in relation to the ice-shelf and sea ice edge. D: Emperor penguins (small black dots) on their way to or from the breeding colony onto of the sea-ice. They have to climb up or down a small ice cliff, which shows up as white in this image. To the left of this cliff is ice shelf, while the ridged area to the right is fast-ice. In this image emperors can be seen on the ice shelf (a long line in upper left and parallel to the cliff face) and the cliff (shorter linear group at middle bottom and perpendicular to the cliff face). The height of this ice cliff is presently unknown but based on the size of the penguins in the image it may be only a few metres high.

However, in 2012, a further satellite survey for emperor colonies was conducted along the edge of the Larsen C Ice Shelf. A medium sized colony was discovered on top of the shelf (at 66.08°S, 60.65°W). An aerial survey of the colony was undertaken in early December 2012 (Figure 3) revealing that the colony comprised around 3,800 adult birds. Archival satellite imagery shows that it has been located on the ice shelf since at least 2008, the earliest imagery available for the area. The Antarctic Peninsula is one of the fastest warming regions [20] and has suffered significant ice shelf loss [21]. The sea-ice regime here has also been affected by climatic forcing and the birds may have moved from the sea-ice creeks to the top of the ice shelf. Exactly how the birds access the shelf is unclear but it appears that they climb the low ice cliff (Figure 4). King penguins climb up dry glaciers in warm weather to stay cool; perhaps the less agile emperor is also able to climb slopes, particularly where ice shelves weather and ablate the steepness of the shelf face.

**Figure 5:**
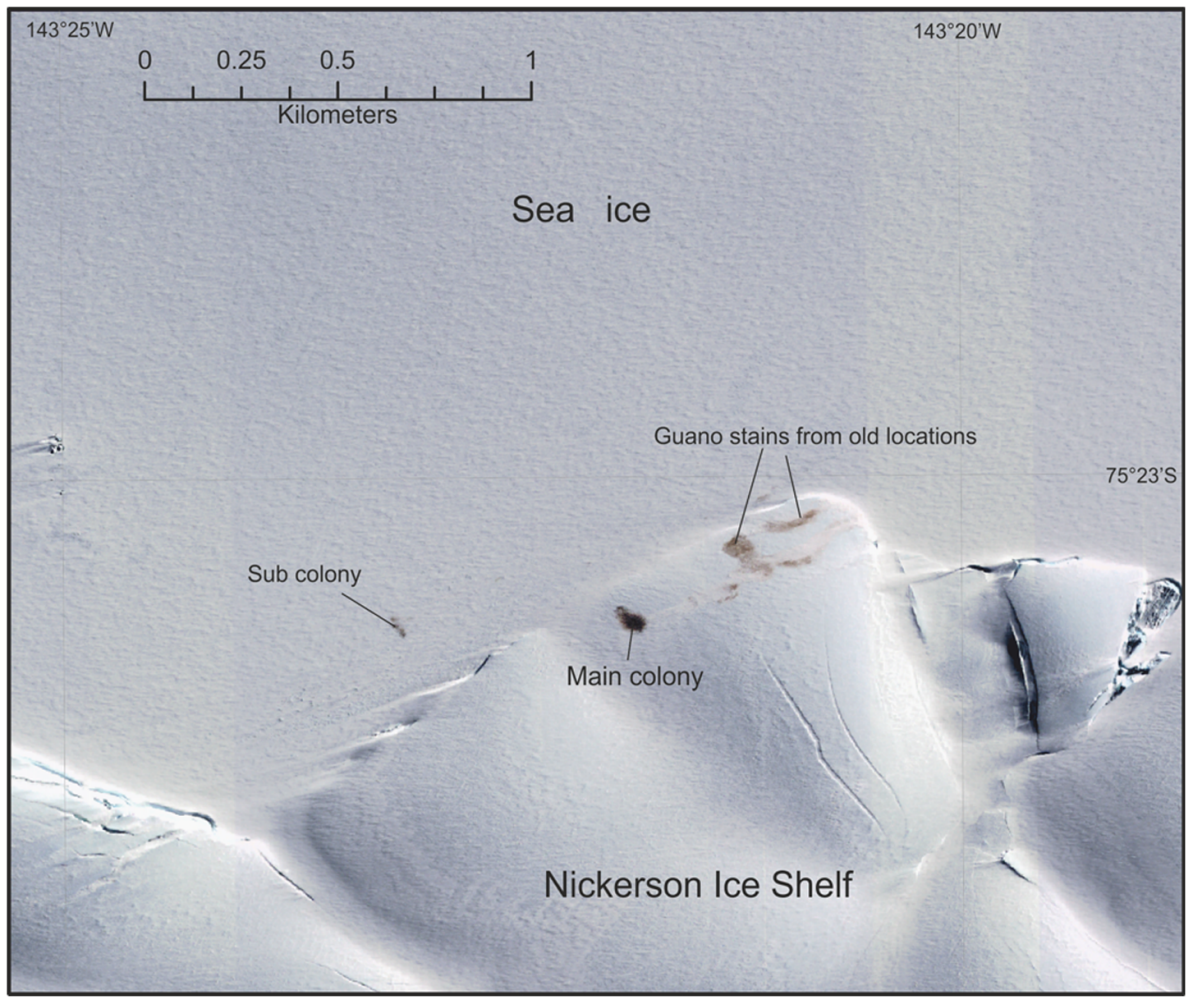
QuickBird 2 very high resolution satellite image of the Ruppert Coast emperor penguin colony(17 October 2012) including part of the Ruppert Coast and Nickerson Ice Shelf, it clearly shows the emperor penguin colony located on the higher ice shelf. Access to the main colony seems to be by a shallow ice ramp north and west of the present location. Brown guano stains mark the previous colony site approximately half a kilometres northeast, and to the west, on the sea-ice is a smaller subgroup of penguins.

Finally, a colony was sighted on top of an ice shelf at the Ruppert Coast colony. This colony was only discovered in 2010 when it was located on the sea-ice under the ice cliffs of the Nickerson Ice Shelf at 75.38°S, 143.35°W. Satellite imagery shows that in 2008, 2011 and 2010 (no images from 2009 are available) the colony was located on the sea-ice at 75.38°S, 143.28°W, but in 2012 (17/10/2012) it had moved onto the edge of the ice shelf above the ice cliff (Figure 5). At present no information is available to suggest why the colony moved onto the shelf in this year.

### Analyses

**Figure 6:**
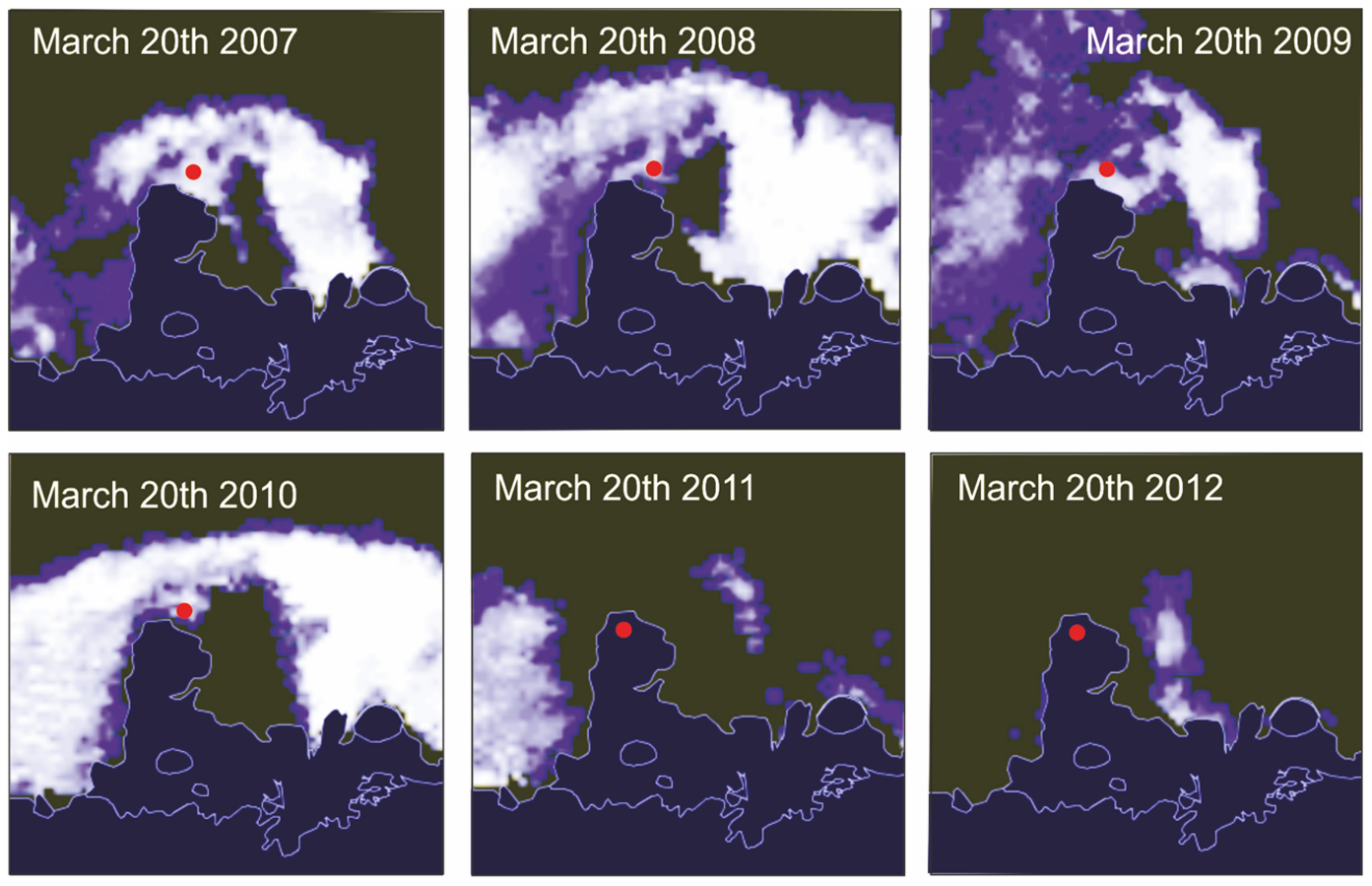
Sea-ice concentration around the Shackleton Ice Shelf for the time period 2007 to 2012, each from the 20^th^ of March. White shading denotes thick sea-ice; blue, thinner sea-ice; black, open water. The red dot shows the location of the breeding colony identified from QuickBird VHR imagery (images courtesy of Polarview/University of Bremen).

To assess why the colony location had moved from sea-ice to ice-shelf ENVISAT synthetic aperture radar imagery of sea-ice concentration was acquired of the Shackleton Ice shelf colony (for which the most data existed) several times over the course of the breeding season. The imagery from March, when adults start to return to their breeding location [22], shows that in 2008, 2009 and 2010 the sea-ice concentration at the initial site was dense and was sufficiently stable for the penguins to access the location (Figure 6). But in 2011 and 2012, the sea-ice did not form until early- to mid-April. The birds therefore chose a site on top of the ice shelf in years when sea-ice formed late. The birds show remarkable fidelity to the site, changing their breeding platform in preference to changing the breeding location when April sea-ice conditions become unsuitable.

**Figure 7:**
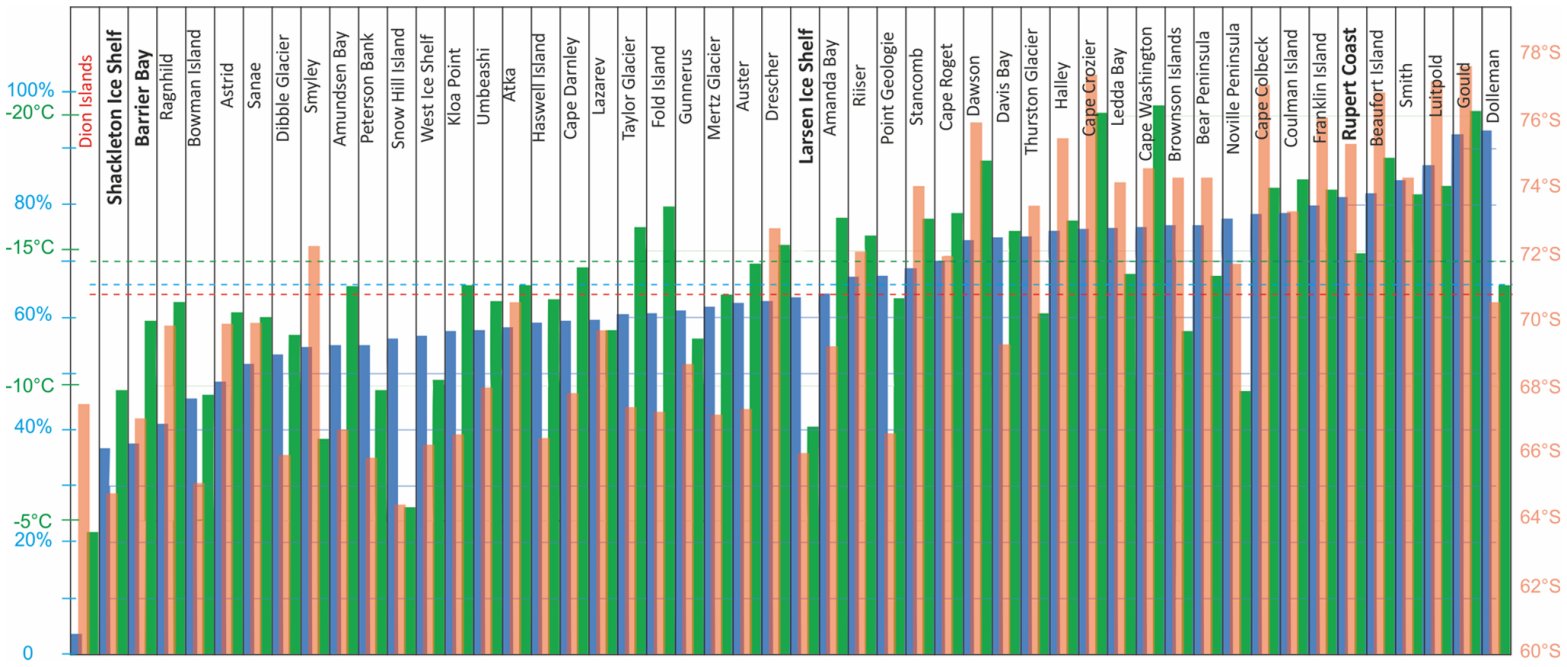
Marginality. Histogram of three parameters at emperor penguin breeding colonies: blue bars represent mean March/April sea-ice concentration, red bars are latitude and green are mean temperature. Colonies are ranked from left to right with lowest sea-ice concentrations to the left. The four colonies mentioned in this paper are named in bold. Dashed lines in blue, red and green denote the mean of the three parameters for all colonies. Shackleton Ice shelf and Barrier Bay colonies are in locations that have the lowest mean autumn sea-ice concentrations. The Larsen ice shelf colony has slightly lower than average sea-ice concentrations, but its latitude and mean annual temperature are well above average. The colony on the far left, Dion Islands, marked in red is believed to have declined and ceased to exist following recent climate change (temperature rise and sea-ice loss) on the Antarctic Peninsula. This previously occupied site is included to give an indication of the current breeding limits. These parameters give no indication of why the Ruppert Coast colony, found breeding on the ice shelf in only one year, relocated onto this ice shelf in 2012. Mean sea-ice concentration (blue) is recorded as a percentage for March/April when emperors prospect and recruit at breeding sites. The figures are calculated using synthetic aperture radar measurements for all dates in these two months for the years 1998–2007. Latitude (red) is the latitude of the colony location from Fretwell et al. 2012 and Wieneke 2012, with the addition of the newly found Larsen Ice Shelf colony described in this paper. Mean temperature is based on a yearly mean for 2000–2004 based on the RACMO climate model.

To test whether the presence of colonies that have been found on ice-shelves was linked to environmental conditions, three environmental variables were assessed at the four sites and compared with values for other emperor penguin colony locations (Figure 7). Autumn (March/April) sea-ice concentration was modelled using synthetic aperture radio imagery from the Polarview website (http://www.polarview.aq/), mean temperature was assessed using the RACMO region climate model [23] and latitude using the recent calculation of the circumpolar emperor penguin population [13].

## Results and Discussion

Of the four colonies described here, the three that have been found breeding on ice shelves in multiple years could be described as located in marginal conditions. The Shackleton Ice Shelf and Barrier Bay sites have the lowest mean autumn sea-ice concentrations of any colonies and both these and the Larsen ice-shelf colony are in the most northerly part of the emperors range and have higher-than-average mean-temperature regimes. Three other colonies have mean annual temperatures higher than the Larsen colony; Smyley Island, Bowman Island and Snow Hill Island. On Smyley Island in 2009 the colony was observed by QuickBird satellite imagery breeding on top of an iceberg (QuickBird catalogue number 101001000A9A8B00 November 12th 2009 ), a possible response to poor sea-ice earlier in the season. A similar behaviour has been observed at the Mertz Glacier colony which is also located in an area that has a high mean temperature (André Ancel pers coms.). This behaviour at these other colonies suggests that breeding on ice-shelves is only one of several possible adaptations that could be employed by emperor penguins when sea-ice conditions are poor; others include moving onto land (for example Dion Islands). Of the other warmer sites the Snow Hill Island colony has not only the warmest mean temperature but is also is the highest latitude, although it has a reasonable high mean sea-ice concentration. Bowman Island has the third highest mean temperature and the fourth lowest sea-ice concentration. These two must be considered some of the most marginal and potentially vulnerable of emperor breeding locations as neither colony has the option of moving onto floating ice-shelves as there are none in the nearby locality and neither has it shown evidence of breeding on icebergs.

The Ruppert Coast colony that has been found located on the edge of an ice shelf in one year is neither in a warm or poor sea-ice location; as yet we have no explanation of why this colony moved to a more elevated location.

Only a relatively small number of emperor colonies have been studied across Antarctica [1], [12], [22], [24], with the longest study based at Pointe Géologie, which has been continually monitored since the 1950s [1], [4], [5], [24]. The behaviour of breeding on ice-shelves, as reported here, has only been reported once at a small colony in East Antarctica [15]. That this behaviour is not the exception but is apparently more common among emperor penguins is a surprising result. The reasons why this behaviour has not been recorded before are unclear, possible explanations include:

1. The phenomenon may be recent phenotypic plasticity as regional climate change affects parts of the Antarctic coastline.
2. Most previous study-sites are not located at ice-shelf breeding colonies where this behaviour is exhibited.
3. There have been no large scale systematic searches for emperors on ice shelves (although many flights and surveys have overflown iceshelves).

Visser [17] has proposed a number of possible adaptations to climate change in birds, including phenotype plasticity, which includes changes in breeding behaviour. One special case of phenotype plasticity noted in the work is that of “learning” where animals can adapt to climate change if they learn from their experiences. Whether the adaptation of emperor penguins breeding on ice-shelves is learnt, or inherited behaviour is at present unclear; the Larsen and Barrier Bay colonies seem to be permanently located on the shelf, but the Shackleton colony moves there only when sea-ice conditions in April dictate. One aspect of Visser’s work that must be considered is that it concentrates on individual nesting birds rather than colonial species. Colonial species, especially emperor penguins, seem to move en-mass to new locations, although how this decision is made is unclear. The previous work may not therefore prove an ideal model for assessing how colonial nesting sea-birds may adapt to climate change.

The ability of emperor penguins to change their breeding platform when fast ice conditions deteriorate may be an important adaptation that could help the species survive in a warming environment. Although regional warming has led to loss of ice-shelves around the Antarctic Peninsula [21] ice-shelves are less sensitive to a warming environment and react to warming on slower timescales than sea-ice, the extent, stability and seasonality of which can change rapidly with warming temperatures as already seen in the Arctic [25], [26], [27], [28] and in the west Antarctic Peninsula [8].

For emperor penguins, the loss of the sea-ice as a breeding platform is not the only consequence of a warming environment. Factors, such as changes to food webs [10], [29] and increased predation and competition [12] will also affect breeding success survival rates and other demographic parameters in areas that experience regional warming. Additionally, there are several negative factors that could potentially result from breeding on ice shelves, including the lack of shelter and exposure to katabatic winds, lack of fresh snow in areas where increased wind speeds scour the surface and a risk from calving ice fronts. How great these risks are and how much of an advantage or disadvantage breeding on ice-shelves proves to be for this species has yet to be quantified. This new discovery also leads to a number of other potential behavioural questions. For example: How do emperors access the ice shelves? Does the breeding cycle differ in locations such as at the Larsen colony where breeding on ice-shelves has become the norm? What is the energetic cost of scaling the ice-front? Such questions will be important future topics of research.

Currently, sea-ice conditions and their predicted decline are one of the key inputs into models that suggest large decreases in emperor penguin numbers. Therefore, the suitability of ice-shelves versus sea-ice as a breeding platform for emperors urgently needs quantifying. Future research efforts need to assess which colonies could potentially move to ice-shelves and whether this newly described behaviour is a temporary or partial solution for coping with future climate change. Emperors are often portrayed as a barometer for the ecosystem, that is, a “canary in the coalmine” for species more difficult to study. This previously unknown and surprising behaviour recorded in such an iconic animal suggests that other species may also be capable of unpredicted or unknown behavioural adaptations that may also increase their survival in a future warming world.
